# Supplementary material for: Intracellular competition for nitrogen controls dinoflagellate population density in corals
Source: Proc Biol Sci. 2020 Mar 4;287(1922):20200049. doi: 10.1098/rspb.2020.0049 (PMC7126079; doi:10.1098/rspb.2020.0049)
Supplement: Supplementary methods, figures, and tables [file rspb20200049supp1.docx]

Supplementary Information for

**Intracellular competition for nitrogen controls dinoflagellate population density in corals**

DOI: 10.1098/rspb.

Krueger T., Horwitz N., Bodin J., Giovani M.-E., Escrig S., Fine M., Meibom A.

Thomas Krueger

Email: tk556@cam.ac.uk

**Contents:**

Supplemental Methods

Figure S1-S4

Table S1-S4

**Supplemental Methods**

*Isotopic hotspot effect in relation to cell diameter*

The large mean symbiont cell diameter (ca. 7-12 µm) makes it unlikely to narrowly miss the presence of cells in the cut 500 nm tissue sections and to underestimate the local tissue density. However, uncertainty associated with obtained symbiont isotopic enrichment arises from the heterogeneous nature of the ^13^C and ^15^N distributions in the symbiont and the effects of cutting cells at different planes. Especially the heterogenous ^13^C-enrichment (manifested as ^13^C-enrichment hotspots in lipid bodies and starch grains) generated a bias. Cutting the cell towards the equator increases the number of sampled starch grains and lipid bodies (^13^C isotopic hotspots), thus increasing the average signal for the cell. Small apparent cell diameters on the other hand create a data bias towards low isotopic enrichment, because only few or no isotopic hotspots that drive the mean enrichment are captured. We have accordingly statistically tested for enrichment bias due to size and excluded symbiont ROIs with small apparent diameters from the isotopic dataset (Fig. S2). Host gastrodermal isotopic enrichment was derived from the tissue adjacent to the symbionts within the picture frame. The movement of photosynthates originating from symbiont cells just outside the image frame should be minimal and/or occur with similar probability in all images. Indeed, the final fitted linear model determined the intercepts of host gastrodermal enrichment for tissue without any symbiont cells to be not significantly different from zero, indicating an absence of host enrichment in the absence of symbiont cells.


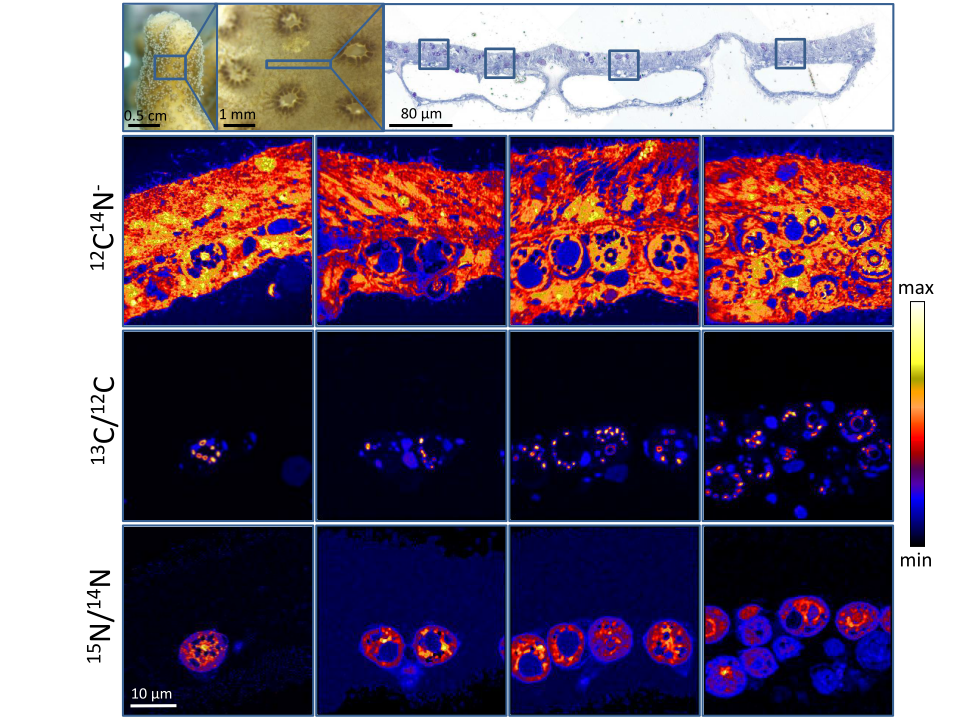


**Figure S1. Illustration of coral nubbins, tissue structures and quantitative NanoSIMS isotopic imaging.** Cross-sections of the polyp-connecting coenenchyme tissue were randomly selected from *Stylophora pistillata* colonies (N=3) incubated with ^13^C-bicarbonate and ^15^N-nitrate for 6 h in the light. NanoSIMS imaging with constant frame-size linked individual symbiont ^13^C- and ^15^N-assimilation to a measure of local cell density (i.e., cells within the image frame) in the gastrodermis.


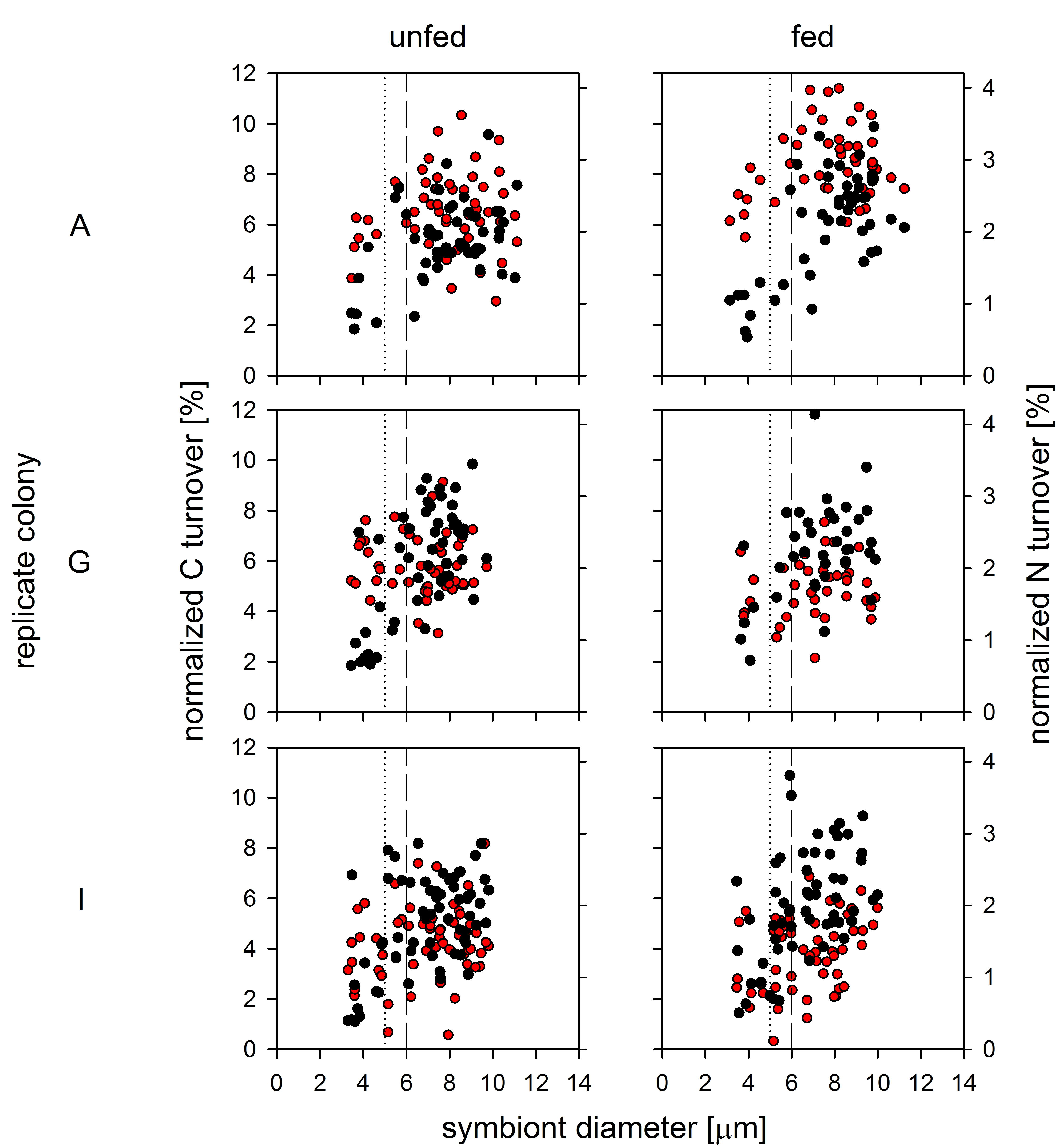


**Figure S2. Relationship between apparent symbiont cell diameter and symbiont nutrient assimilation in three colonies of *Stylophora pistillata* as derived from NanoSIMS image analysis.** Data show autotrophic carbon (black, left axis) and nitrogen turnover (red, right axis) of individual symbiont cells in relation to their apparent cell diameter for both feeding treatments. Due to the size-related enrichment bias (see Methods), the complete dataset shown here was reduced by excluding cells smaller than 5 µm for the nitrogen dataset (threshold as dotted line) and cells smaller than 6 µm for the carbon dataset (dashed line) prior to final analysis. Above these thresholds, there is no significant cell-size bias in the NanoSIMS data.


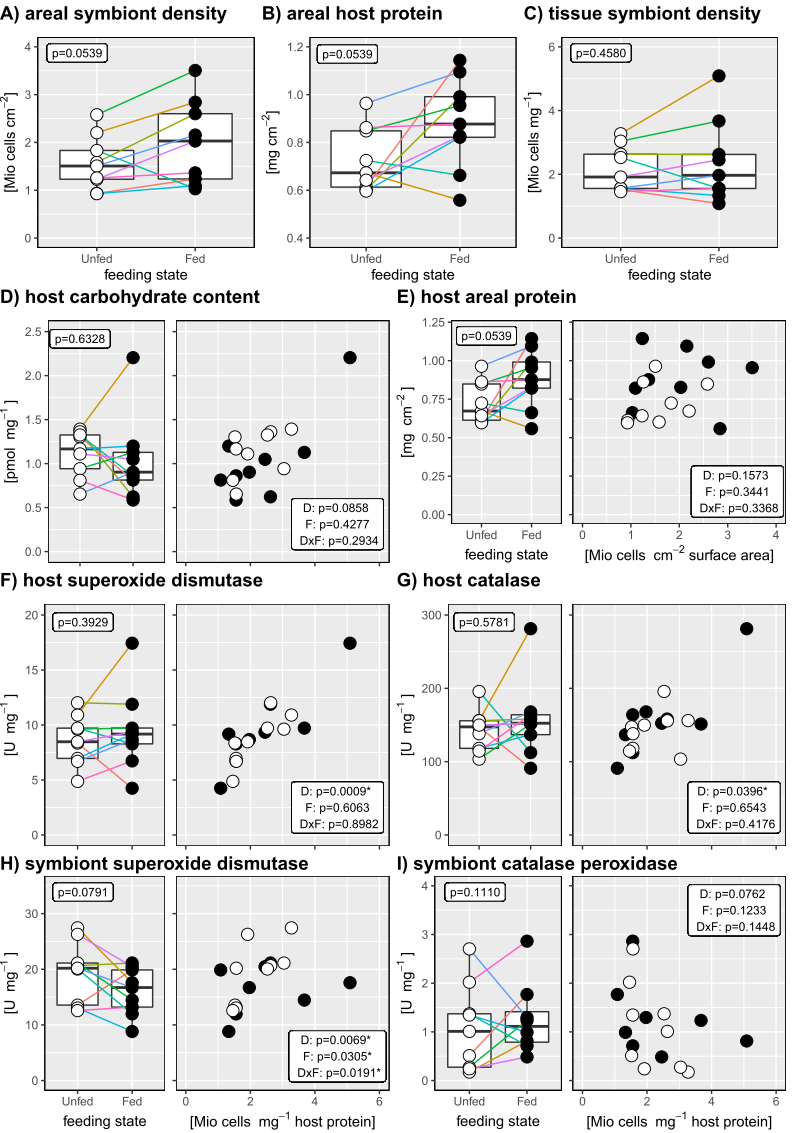


[previous page] **Figure S3. The effect of regular feeding and symbiont density on host biomass and antioxidants.** All graphs show physiological variables in unfed (white) and regularly fed fragments (black) of paired fragments from the same mother colonies (colours). (A-C) Increase in symbiont and host biomass due to feeding as isometric effect with unaltered tissue symbiont density (D) host carbohydrate content per milligram of host protein, (E) relationship between symbiont areal density and host areal protein content; note different unit on x-axis, (F-G) host enzymatic antioxidants, (H-I) symbiont enzymatic antioxidants. Shown statistical details refer to the average feeding effect (boxplots; Table S1) and the effects of density (D) and feeding (F) (Table S2).


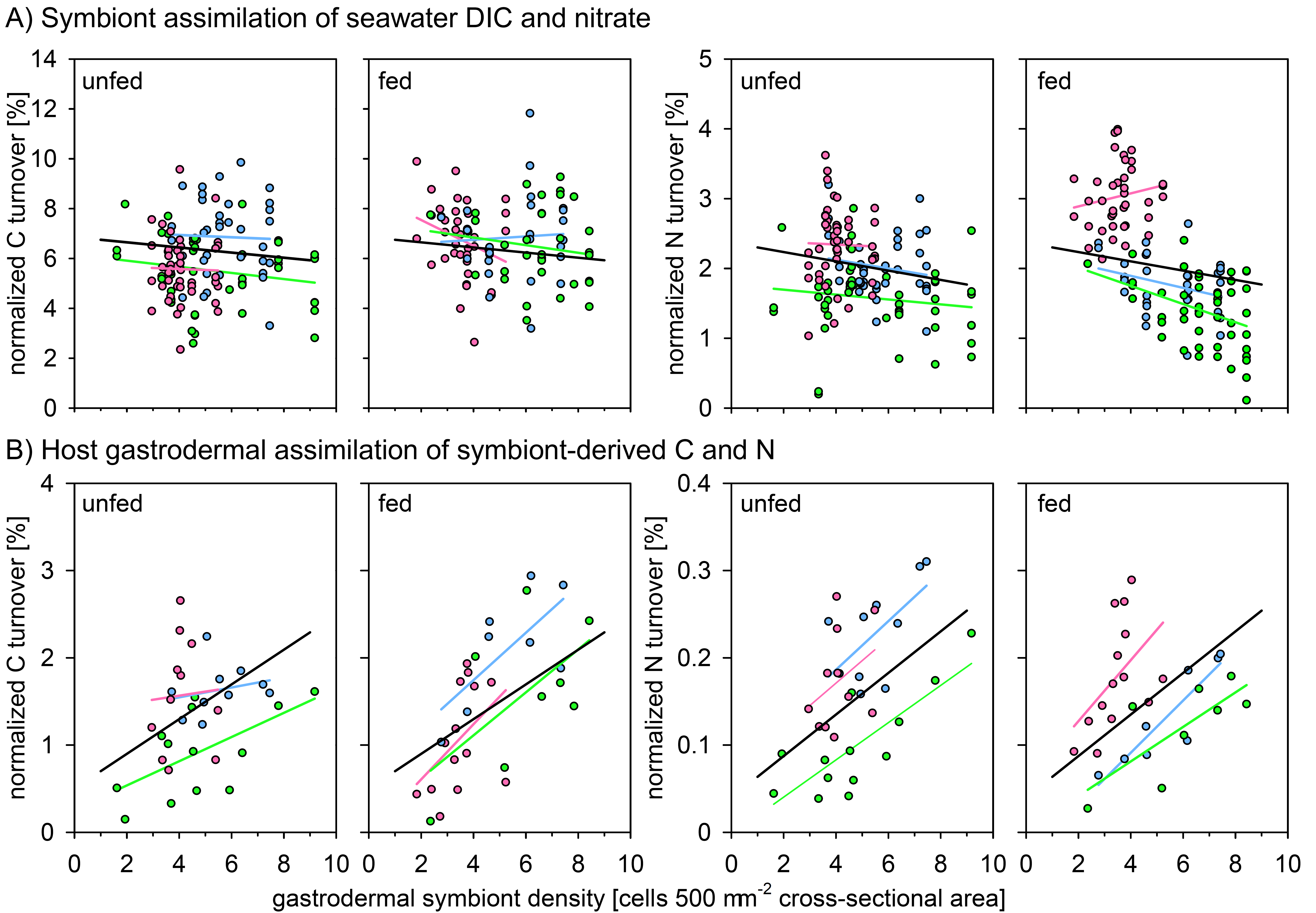


**Figure S4. Colony-specific responses between local coral tissue symbiont density and autotrophic carbon and nitrogen assimilation.** Data plots show the resolved raw data for symbiont and host depicted in Fig. 4 and 5. Relative structural carbon and nitrogen turnover from autotrophy in (A) individual symbionts and (B) surrounding host gastrodermis in relation to local symbiont density in gastrodermal cross-sections after incubation with ^13^C DIC and ^15^N nitrate for 6 h in the light. Colony-specific ordinary least square regressions of raw data (coloured lines) are shown in addition to overall model fit (black line). Note that the overall regression in (B) is depicted including the intercept for illustration purposes. The intercept is in fact not statistically significantly different from zero (Fig. 4, Table S3).

**Table S1. Statistical output of pairwise three-way ANOVA for the effect of regular feeding on various biometric and physiological coral properties.** Degrees of freedom (df) are indicated for each factor. The type of normalization for each variable (per symbiont cell; per unit protein; per coral surface area) is shown in brackets (cf. Fig. 2, S1). Asterisks indicate significance at p ≤ 0.05 with N=9, except where the pairs for outlier colonies E, F where removed for oxygen metabolism (^‡^) (see Fig. S1G) and N=8 for symbiont protein and carbohydrate due to missing value. Effect sizes are shown as means of relative pairwise differences between paired replicates including confidence intervals.

| **variable** | **feeding (F) (df=1)** | **replicate [replicate tank] (df=6)** | **replicate tank (df=2)** | **data transformation** | **pairwise difference (95%CI)** | **figure** | **comments** |
| --- | --- | --- | --- | --- | --- | --- | --- |
| F_v_/F_m_ | F = 8.577 | F = 4.605 | F = 4.222 | none | +2.4% (0.5-4.4%) | Fig. 2A |  |
|  | p = 0.0190* | p = 0.0257* | p = 0.0560 |  |  |  |  |
| rETRmax | F = 8.266 | F = 3.109 | F = 8.055 | none | +24% (4.7-43.1%) | Fig. 2B |  |
|  | p = 0.0207* | p = 0.0706 | p = 0.0121* |  |  |  |  |
| rα | F = 0.164 | F = 2.665 | F = 0.784 | none | -1.3% (-14.1-+11.4%) | Fig. 2C |  |
|  | p = 0.6961 | p = 0.1003 | p = 0.4888 |  |  |  |  |
| I_k_ | F = 6.775 | F = 1.271 | F = 2.635 | none | +29% (3.5-53.9%) | Fig. 2D |  |
|  | p = 0.0315* | p = 0.3665 | p = 0.1321 |  |  |  |  |
| Total Chl (cell) | F = 24.384 | F = 1.218 | F = 7.546 | none | +28% (10.5-45.2%) | Fig. 2E |  |
|  | p = 0.0011* | p = 0.3869 | p = 0.0144* |  |  |  |  |
| Total Chl (surface) | F = 11.629 | F = 4.756 | F = 1.599 | none | +66% (22.2-110.4%) | not shown |  |
|  | p = 0.0092* | p = 0.0235* | p = 0.2605 |  |  |  |  |
| Chl *a*/chl *c* | F = 1.460 | F = 2.706 | F = 41.763 | LOG | -3.5% (-11.5-+4.5%) | Fig. 2F |  |
|  | p = 0.2614 | p = 0.0969 | p < 0.0001* |  |  |  |  |
| ^‡^P_gross_ symbiont (cell) | F = 88.143 | F = 2.357 | F =10.815 | LOG | -32.7% (-39.7-25.6%) | Fig. 2G | without colony E,F |
|  | p < 0.0001* | p = 0.1667 | p = 0.0102* |  |  |  |  |
| ^‡^P_gross_ (Chl) | F = 65.703 | F = 1.086 | F = 0.562 | LOG | -47.4% (-57.4-37.4%) | not shown | without colony E,F |
|  | p < 0.0001* | p = 0.4411 | p = 0.5975 |  |  |  |  |
| ^‡^P_gross_ holobiont (protein) | F = 12.583 | F = 21.483 | F = 17.128 | none | -23.2% (-37.1-9.3%) | Fig. 2H | without colony E,F |
|  | p = 0.0121* | p = 0.0011* | p = 0.0033* |  |  |  |  |
| ^‡^P_gross_ (surface) | F = 0.005 | F = 8.444 | F = 4.341 | none | -6.8% (-15.8-+2.2%) | not shown | without colony E,F |
|  | p = 0.9436 | p = 0.0041* | p = 0.0529 |  |  |  |  |
| Holobiont respiration (protein) | F = 3.285 | F = 4.264 | F = 3.232 | none | -11% (-25.3-+3.9%) | not shown |  |
|  | p = 0.1057 | p = 0.0317* | p = 0.0936 |  |  |  |  |
| Holobiont respiration (surface) | F = 0.289 | F = 4.026 | F = 0.812 | none | +7.1% (-7.1-+21.2%) | not shown |  |
|  | p = 0.6057 | p = 0.0370* | p = 0.4773 |  |  |  |  |
| ^‡^P_net_ holobiont (protein) | F = 7.161 | F = 12.430 | F = 9.347 | SQRT | -28.6% (-56.9-0.3%) | not shown | without colony E,F |
|  | p = 0.0367* | p = 0.0046* | p = 0.0143* |  |  |  |  |
| ^‡^P_net_ (surface) | F = 2.041 | F = 10.089 | F = 7.802 | none | -15.4% (-41.5-+10.7%) | not shown | without colony E,F |
|  | p = 0.2030 | p = 0.0078* | p = 0.0214* |  |  |  |  |
| ^‡^daily P_gross_:R | F = 1.910 | F = 5.149 | F = 4.207 | none | -7.6% (-25.8-+10.5%) | not shown | without colony E,F |
|  | p = 0.2162 | p = 0.0382* | p = 0.0721 |  |  |  |  |
| ^‡^Light R:P_gross_ | F = 1.850 | F = 3.228 | F = 2.023 | none | +11.5% (-5.1-+28.1%) | not shown | without colony E,F |
|  | p = 0.223 | p = 0.0973 | p = 0.2131 |  |  |  |  |
| Symbiont density (surface) | F = 5.100 | F = 7.444 | F = 0.995 | none | +28% (2.8-53.2%) | Fig. S2A |  |
|  | p = 0.0539 | p = 0.0062* | p = 0.4112 |  |  |  |  |
| Symbiont density (biomass) | F = 0.608 | F = 6.758 | F = 3.772 | none | +6.1% (-17.0-+29.2%) | Fig. S2C |  |
|  | p = 0.4580 | p = 0.0083* | p = 0.0702 |  |  |  |  |
| Symbiont protein (cell) | F = 0.548 | F = 13.522 | F = 0.673 | none | -0.2% (-20.3-+20.0%) | Fig. 3 | without B; B fed missing |
|  | p = 0.4833 | p = 0.0018* | p = 0.5405 |  |  |  |  |
| Symbiont Carbs (cell) | F = 0.194 | F = 9.120 | F = 1.265 | none | -8.0% (-32.9-+16.9%) | Fig. 3 | without B; B fed missing |
|  | p = 0.6732 | p = 0.0057* | p = 0.3395 |  |  |  |  |
| Host protein (surface) | F = 5.097 | F = 1.650 | F = 1.167 | none | +25% (-1.7-+50.6%) | Fig. S2B, E |  |
|  | p = 0.0539 | p = 0.2502 | p = 0.3593 |  |  |  |  |
| Host Carbs (protein) | F = 0.247 | F = 1.494 | F = 2.558 | none | -4.6% (-33.6-+24.5%) | Fig. S2D |  |
|  | p = 0.6328 | p = 0.2921 | p = 0.1384 |  |  |  |  |

| Host SOD (protein) | F = 0.816 | F = 3.078 | F = 3.985 | none | +12% (-13.2-+36.7%) | Fig. S2F |  |
| --- | --- | --- | --- | --- | --- | --- | --- |
|  | p = 0.3929 | p = 0.0723 | p = 0.0630 |  |  |  |  |
| Host CAT (protein) | F = 0.336 | F = 0.911 | F = 0.5291 | LOG | +14% (-16.1-+44.9%) | Fig. S2G |  |
|  | p = 0.5781 | p = 0.5321 | p = 0.6084 |  |  |  |  |
| Symbiont SOD (protein) | F = 4.044 | F = 2.358 | F = 3.145 | none | -14% (-35.3-+7.46%) | Fig. S2H |  |
|  | p = 0.0791 | p = 0.1114 | p = 0.0982 |  |  |  |  |
| Symbiont KatG (protein) | F = 3.210 | F = 3.003 | F = 0.330 | LOG | +142% (7.9-275.3%) | Fig. S2I |  |
|  | p = 0.1110 | p = 0.0765 | p = 0.7284 |  |  |  |  |

**Table S2. Quantifying the relationship between symbiont density and individual symbiont biomass and nutrient assimilation under fed and unfed conditions.** (A) Orthogonal regression fits for transformed negative correlations between coral tissue symbiont density and symbiont soluble protein and carbohydrate content (as shown in Fig. 2). Relative change indicates density effect for the observed maximal density range (1.1-3.7 Mio. cells mg^-1^ host protein). Unit for density is Mio. cells mg^-1^ host protein; symbiont protein and carbohydrate content in pg cell^-1^ with BSA and glucose as reference molecules. Note SQRT transformation of x and y due to hyperbolic relationship (Fig. 3 inserts) (B-D) Linear mixed model estimates for effects of local symbiont density on (B) anabolic turnover of photoautotrophic C and N in symbiont, (C) on individual symbiont turnover of heterotrophic C and N input, and (D) assimilation and turnover of translocated photosynthates in surrounding host gastrodermis (model details in Table S4). Slopes and intercepts as source-normalized atom percent excess (% APE; see Methods) relative to local tissue density in cells per 500 µm^2^ cross-sectional gastrodermal area (cf. Figs. 4-5). Absolute changes (as percentage points) and relative changes (as percentage) are reported as the observed local symbiont abundance increases from 1 to 9 cells per 500 µm^2^ cross-sectional gastrodermal area (cf. Figs. 4-5). Asterisks indicate whether estimates for slopes and intercepts are statistically significantly different from zero (Table S4).

(A) symbiont soluble protein and carbohydrate content

| relationship (x *vs*. y) | feeding acclimation | intercept [pg cell^-1^] | slope | lower/upper 95%CL | relative change |
| --- | --- | --- | --- | --- | --- |
| SQRT density *vs*. SQRT symbiont protein | unfed | 7.95* | -3.599* | -8.278/-1.564 |  |
|  | fed | 5.91* | -2.243* | -3.644/-1.380 |  |
|  | **overall fit** | **6.89*** | **-2.902*** | **-4.333/-1.943** | **-88.1%** |
| SQRT density *vs*. SQRT symbiont carbohydrates | unfed | 3.26* | -1.385* | -2.540/-0.756 |  |
|  | fed | 3.11* | -1.333* | -1.780/-0.998 |  |
|  | **overall fit** | **3.17*** | **-1.348*** | **-1.747/-1.040** | **-89.2%** |

(B) symbiont autotrophic assimilation

| element | feeding acclimation | intercept [%APE] | slope | \| change from 1 to 9 cells \| \| \| --- \| --- \| \| absolute \| relative \| | |
| --- | --- | --- | --- | --- | --- | --- | --- | --- | --- |
| ^13^C | unfed | 6.489 | -0.1026 |  |  |
|  | fed | 7.221 | -0.1026 |  |  |
|  | **overall fit** | **6.854*** | **-0.1026** | **n.s.** | **n.s.** |
| ^15^N | unfed | 2.320 | -0.0663 |  |  |
|  | fed | 2.416 | -0.0663 |  |  |
|  | **overall fit** | **2.368*** | **-0.0663*** | **-0.5304** | **-23%** |

[table S2 continued]

(C) symbiont heterotrophic assimilation

| element | feeding acclimation | intercept [%APE] | slope | \| change from 1 to 9 cells \| \| \| --- \| --- \| \| absolute \| relative \| | |
| --- | --- | --- | --- | --- | --- | --- | --- | --- | --- |
| ^13^C | fed | **2.296*** | **-0.0284** | **n.s.** | **n.s.** |
| ^15^N | fed | **5.710*** | **-0.2146*** | **-1.717** | **-31%** |

(D) host autotrophic assimilation

| element | feeding acclimation | intercept [%APE] | slope | \| change from 1 to 9 cells \| \| \| --- \| --- \| \| absolute \| relative \| | |
| --- | --- | --- | --- | --- | --- | --- | --- | --- | --- |
| ^13^C | unfed | 0.4254 | +0.1990 |  |  |
|  | fed | 0.5766 | +0.1990 |  |  |
|  | **overall fit** | **0.5010** | **+0.1990*** | **+1.5920** | **+800%** |
| ^15^N | unfed | +0.0528 | +0.0238 |  |  |
|  | fed | +0.0268 | +0.0238 |  |  |
|  | **overall fit** | **0.0398** | **+0.0238*** | **+0.1904** | **+800%** |

**Table S3. Statistical output of ANCOVA on the effects of symbiont density on physiological variables in dependence of feeding state and accounting for tank replication.** Degrees of freedom (df) are indicated for each factor. Symbiont densities (in cells mg^-1^ host protein) was used as continuous predictor for all variables except host areal protein content (^‡^), where the unit of the predictor was changed to cells cm^-2^ (cf. Fig. S2E). The type of normalization for each variable (per symbiont cell; per mg host protein) is shown in brackets. Asterisks indicate significance at p≤0.05. N=9, except for some oxygen-related variables (^†^), where N = 7 in the feeding treatment due to removal of outlier colonies E, F (cf. Fig 2G, H).

|  | **density (df=1)** | **feeding (df=1)** | **density x feeding (df=1)** | **tank (df=2)** | **transformation** | **figure** |
| --- | --- | --- | --- | --- | --- | --- |
| F_v_/F_m_ | F = 11.717, p = 0.0050* | F = 3.635, p = 0.0808 | F = 3.137, p = 0.1019 | F = 0.223, p = 0.8033 | none | Fig. 2A |
| rETRmax | F = 5.405, p = 0.0384* | F = 4.301, p = 0.0603 | F = 0.150, p = 0.7055 | F = 2.142, p =0.1602 | none | Fig. 2B |
| rα | F = 1.585, p = 0.2320 | F = 0.165, p = 0.6915 | F = 0.816, p = 0.3843 | F = 0.273, p = 0.7659 | ^3 | Fig. 2C |
| I_k_ | F = 0.771, p = 0.3971 | F = 5.085, p = 0.0436* | F = 0.003, p = 0.9558 | F = 1.178, p = 0.3411 | none | Fig. 2D |
| Total Chl (cell) | F = 7.853, p = 0.0160* | F = 27.153, p = 0.0002* | F = 1.706, p = 0.2159 | F = 10.351, p = 0.0024* | none | Fig. 2E |
| Chl *a*/chl *c_2_* | F = 24.608, p = 0.0003* | F = 0.666, p = 0.4305 | F = 4.453, p = 0.0565 | F = 67.510, p < 0.0001* | LOG | Fig. 2F |
| ^†^P_gross_ symbiont (cell) | F = 2.458, p = 0.1480 | F = 3.805, p = 0.0797 | F = 0.020, p = 0.8903 | F = 1.357, p = 0.3009 | LOG | Fig. 2G |
| ^†^P_gross_ holobiont (protein) | F = 16.660, p = 0.0022* | F = 5.692, p = 0.0382* | F = 1.829, p = 0.2060 | F = 0.546, p = 0.5955 | none | Fig. 2H |
| Holobiont respiration (protein) | F = 14.380, p = 0.0035* | F = 6.800, p = 0.0261* | F = 2.778, p = 0.1265 | F = 0.888, p = 0.4415 | none | not shown |
| ^†^P_net_ holobiont (protein) | F = 9.233, p = 0.0125* | F = 3.739, p = 0.0820 | F = 0.279, p = 0.6089 | F = 0.582, p = 0.5767 | SQRT | not shown |
| Host Carbs (protein) | F = 3.505, p = 0.0858 | F = 0.674, p = 0.4277 | F = 1.207, p = 0.2934 | F = 1.198, p = 0.3354 | none | Fig. S2D |
| ^‡^Host protein (surface) | F = 2.276, p = 0.1573 | F = 1.167, p = 0.3441 | F = 1.001, p = 0.3368 | F = 0.750, p = 0.4036 | none | Fig. S2E |
| Host SOD (protein) | F = 19.254, p = 0.0009* | F = 0.280, p = 0.6063 | F = 0.017, p = 0.8982 | F = 0.892. p = 0.4353 | none | Fig. S2F |
| Host CAT (protein) | F = 5.327, p = 0.0396* | F = 0.211, p = 0.6543 | F = 0.705, p = 0.4176 | F = 0.763, p = 0.4874 | LOG | Fig. S2G |
| Symbiont SOD (protein) | F = 10.603, p = 0.0069* | F = 6.010, p = 0.0305* | F = 7.329, p = 0.0191* | F = 4.125. p = 0.0433* | none | Fig. S2H |
| Symbiont KatG (protein) | F = 3.765, p = 0.0762 | F = 2.747, p = 0.1233 | F = 2.432, p = 0.1448 | F = 0.060, p = 0.9423 | LOG | Fig. S2I |

**Table S4. Statistical models.** Mixed linear models for the effects of local density on anabolic turnover of (A) photoautotrophic C and N in individual symbionts, (B) turnover of heterotrophic C and N in individual symbionts, and (C) turnover of translocated photoautotrophic C and N in the surrounding host gastrodermal tissue. Units of local symbiont density (# of symbionts) is in cells per 500 µm^2^ cross-sectional gastrodermal area and intercepts are in source-normalized atom percent excess (in %) as derived from NanoSIMS image analysis (see Methods). Estimates for each fixed factor with upper and lower confidence limit (CL) and cumulative percentage of variance component estimates (VCE) for random factors are indicated.

(A)

| **model** | **fixed factors** | **test-ratio** | **estimate** | **lower CL** | **upper CL** |
| --- | --- | --- | --- | --- | --- |
| ^13^C | intercept | t = 14.00, p<0.0001* | 6.854 | 5.762 | 7.946 |
| R^2^_adj_=13.8% | # of symbionts (N) | *F*_1,159.8_ = 1.848, p=0.1759 | -0.1026 | -0.2516 | 0.0464 |
| N=221 | feeding acclimation (F) | *F*_1,1.874_ = 3.595, p=0.2069 | ±0.3665 | -1.2543 | 0.5213 |
| for cells <6 µm | N x F | *F*_1,77.35_ = 0.0673, p=0.7961 | ±0.0198 | -0.132 | 0.1715 |
|  | **random factors** | **REML VCE** |  |  |  |
|  | replicate (R) | 5.9% |  |  |  |
|  | R x F | 6.4% |  |  |  |
|  | Residual | 87.7% |  |  |  |
| ^15^N | intercept | t = 7.32, p=0.0085* | 2.368 | 1.239 | 3.496 |
| R^2^_adj_=56.5% | # of symbionts (N) | *F*_1,250.7_ = 8.890, p=0.0031* | -0.0663 | -0.1101 | -0.0225 |
| N=255 | feeding acclimation (F) | *F*_1,876_ = 0.148, p=0.7394 | ±0.0477 | -0.6158 | 0.5204 |
| for cells <5 µm | N x F | *F*_1,227_ = 1.756, p=0.1865 | ±0.0298 | -0.0145 | 0.074 |
|  | **random factors** | **REML VCE** |  |  |  |
|  | replicate (R) | 41.2% |  |  |  |
|  | R x F | 15.7% |  |  |  |
|  | Residual | 43.1% |  |  |  |

[table S4 continued]

(B)

| **model** | **fixed factors** | **test-ratio** | **estimate** | **lower CL** | **upper CL** |
| --- | --- | --- | --- | --- | --- |
| ^13^C | intercept | t = 4.07, p=0.0340* | 2.296 | 0.354 | 4.239 |
| R^2^_adj_=60.7% | # of symbionts (N) | *F*_1,94.71_ = 0.315, p=0.5760 | -0.0284 | -0.129 | 0.0722 |
| N=97 | **random factors** | **REML VCE** |  |  |  |
| for cells <6 µm | replicate (R) | 69.3% |  |  |  |
|  | Residual | 30.7% |  |  |  |
| ^15^N | intercept | t = 5.00, p=0.0253* | 5.710 | 1.525 | 9.895 |
| R^2^_adj_=72.1% | # of symbionts (N) | *F*_1,112.4_ = 6.486, p=0.0122* | -0.2146 | -0.3815 | -0.0476 |
| N=115 | **random factors** | **REML VCE** |  |  |  |
| for cells <5 µm | replicate (R) | 73.3% |  |  |  |
|  | Residual | 26.7% |  |  |  |

(C)

| **model** | **fixed factors** | **test-ratio** | **estimate** | **lower CL** | **upper CL** |
| --- | --- | --- | --- | --- | --- |
| ^13^C | intercept | t = 1.63, p=0.1402 | 0.5010 | -0.204 | 1.206 |
| R^2^_adj_=45.1% | # of symbionts (N) | *F*_1,59.9_ = 17.435, p<0.0001* | 0.1990 | 0.104 | 0.294 |
| N=64 | feeding acclimation (F) | *F*_1,1.592_ = 0.566, p=0.5473 | ±0.0756 | -0.633 | 0.482 |
|  | N x F | *F*_1,44.53_ = 3.591, p=0.0646 | ±0.0930 | -0.192 | 0.006 |
|  | **random factors** | **REML VCE** |  |  |  |
|  | replicate (R) | 22.0% |  |  |  |
|  | R x F | 8.0% |  |  |  |
|  | Residual | 70.0% |  |  |  |
| ^15^N | intercept | t = 1.08, p=0.3436 | 0.0398 | -0.065 | 0.1449 |
| R^2^_adj_=61.0% | # of symbionts (N) | *F*_1,59.03_ = 35.323, p<0.0001* | 0.0238 | 0.0158 | 0.0318 |
| N=64 | feeding acclimation (F) | *F*_1,1.925_ = 0.556, p=0.5361 | ±0.0130 | -0.0648 | 0.0907 |
|  | N x F | *F*_1,58.35_ = 0.011, p=0.9181 | ±0.0004 | -0.0085 | 0.0077 |
|  | **random factors** | **REML VCE** |  |  |  |
|  | replicate (R) | 34.9% |  |  |  |
|  | R x F | 28.4% |  |  |  |
|  | Residual | 36.7% |  |  |  |
